# Enhanced Removal of a Pesticides Mixture by Single Cultures and Consortia of Free and Immobilized *Streptomyces* Strains

**DOI:** 10.1155/2013/392573

**Published:** 2013-06-20

**Authors:** María S. Fuentes, Gabriela E. Briceño, Juliana M. Saez, Claudia S. Benimeli, María C. Diez, María J. Amoroso

**Affiliations:** ^1^Planta Piloto de Procesos Industriales Microbiológicos (PROIMI-CONICET), Avenida Belgrano y Pasaje Caseros, 4000 Tucumán, Argentina; ^2^Núcleo de Desarrollo Científico Tecnológico, Universidad de La Frontera, Avenida Francisco Salazar 01145, 4780000 Temuco, Chile; ^3^Departamento de Ingeniería Química, Universidad de La Frontera, Avenida Francisco Salazar 01145, 4780000 Temuco, Chile; ^4^Universidad del Norte Santo Tomás de Aquino, 9 de Julio 165, 4000 Tucumán, Argentina; ^5^Unidad de Administración Territorial, Centro Científico Tecnológico, CONICET-Tucumán, Crisóstomo Álvarez 722, 4000 Tucumán, Argentina; ^6^Facultad de Bioquímica, Química y Farmacia, Universidad Nacional de Tucumán, Ayacucho 491, 4000 Tucumán, Argentina

## Abstract

Pesticides are normally used to control specific pests and to increase the productivity in crops; as a result, soils are contaminated with mixtures of pesticides. In this work, the ability of *Streptomyces* strains (either as pure or mixed cultures) to remove pentachlorophenol and chlorpyrifos was studied. The antagonism among the strains and their tolerance to the toxic mixture was evaluated. Results revealed that the strains did not have any antagonistic effects and showed tolerance against the pesticides mixture. In fact, the growth of mixed cultures was significantly higher than in pure cultures. Moreover, a pure culture (*Streptomyces* sp. A5) and a quadruple culture had the highest pentachlorophenol removal percentages (10.6% and 10.1%, resp.), while *Streptomyces* sp. M7 presented the best chlorpyrifos removal (99.2%). Mixed culture of all *Streptomyces* spp. when assayed either as free or immobilized cells showed chlorpyrifos removal percentages of 40.17% and 71.05%, respectively, and for pentachlorophenol 5.24% and 14.72%, respectively, suggesting better removal of both pesticides by using immobilized cells. These results reveal that environments contaminated with mixtures of xenobiotics could be successfully cleaned up by using either free or immobilized cultures of *Streptomyces*, through *in situ* or *ex situ* remediation techniques.

## 1. Introduction

The agriculture sector is a very important part of the economy and provides foods and raw materials needed for a sustainable development. For this reason, this sector uses different resources as pesticides, chemical fertilizers, equipment, and machines [1]. Pesticides are usually applied simultaneously or one after another for crop protection, and this type of pesticide application often leads to a combined contamination of these compound residues in the soil environment [2]. Among the pesticides, compounds such as organochlorines, organophosphates, carbamates, and pyrethroids are commonly used in vegetables and other crops, in order to increase the productivity [3]. As a result, an increase in the pesticide application translates into an increase in their residues in all spheres of environment [4], especially in agricultural soils. 

Because of the restriction imposed on toxic organophosphate compounds, chlorpyrifos (CP), a broad-spectrum and moderately toxic organophosphate insecticide, has gained the status of one of the most widely used commercial compounds [5]. The widespread use of this pesticide and its resulting residues, which accumulates on agricultural crops, produces an increase of not only environmental contamination but also health hazards to consumers [6]. Moreover, it has been determined that CP resistance to biodegradation is increased due to the accumulation of one of its main byproducts known as 3,5,6-trichloro-2-pyridinol (TCP). TCP is listed as a persistent and mobile pollutant by the US Environmental Protection Agency [7] and shows relatively high antimicrobial effects on microorganisms, which prevents its own degradation [8]. The contamination of soil by CP can be caused by the handling of the pesticide in the farmyard and by the rinsing of containers [6]. CP, among others pesticides, has been detected in groundwater in Greece: 0.005–0.01 *μ*g L^−1^, and in Brazil in superficial water, rivers, and lakes: 0.001–0.174 *μ*g L^−1^ [9].

Other substances widely used in both agricultural and industrial sector are chlorophenolic compounds, which are applied as broad spectrum biocides [10]. Among them, pentachlorophenol (PCP) and its sodium salt have been used as wood and leather preservatives [11]. PCP is toxic to all life forms since it inhibits oxidative phosphorylation [12]. Furthermore, it can accumulate in living organisms and thus produce adverse effect as carcinogenicity and acute toxicity [13]. In addition, this compound is highly recalcitrant due to the stability of the aromatic ring and its high degree of chlorination [14], whereby it can be considered as a hazardous pollutant for the environment. In surface waters from different countries, PCP was detected at concentrations ranging from trace levels to 10,500 *μ*g L^−1^ [13]. In Chile, it was found in water samples of the Limari river basin [9].

Thus, remediation of CP and PCP-contaminated sites is urgently required. A number of methods, including chemical treatment, volatilization, photodecomposition, incineration, and stockpiling, can be applied for the detoxification of these xenobiotic compounds [15–17]. However, most of them are not applicable for diffused contamination at low concentration making it expensive, slow, inefficient, and not always environmental friendly. Thus, biotic degradation is one of the most viable options for the remediation of CP and PCP in soil and water.

In some early studies, CP was reported to be resistant to biodegradation due to accumulation of the antimicrobial degradation products in soil [18]. However, subsequent studies have revealed that many microorganisms are capable of degrading CP efficiently [19–22]. Moreover, different researchers have reported PCP degrading microorganisms from the natural environment. Several bacterial strains such as *Arthrobacter, Pseudomonas, Sphingobium chlorophenolicum,* and *Serratia marcescens* capable of PCP degradation have been reported [23–26]. Among Gram-positive microorganisms, actinobacteria have a great potential for biodegradation of organic and inorganic toxic compounds, and previous studies demonstrated the ability of different genera of actinobacteria to degrade pesticides such as lindane, chlordane, methoxychlor, CP, diuron, and PCP [6, 27, 28].

Complete mineralization of pesticides or their transformation to nontoxic products is desirable in bioremediation process, which is more reasonable with the use of microbial consortia rather than single cultures [29]. Microbial consortia have been shown to be more suitable for bioremediation of recalcitrant compounds as their biodiversity supports environmental survival and increase the number of catabolic pathways available for contaminant biodegradation [30]. However, there are no reports about simultaneous bioremediation of CP and PCP, particularly by actinobacteria consortia. Thus, the aim of this work was to evaluate the ability of pure as well as mixed actinobacteria cultures isolated from different contaminated environments, to degrade a pentachlorophenol and chlorpyrifos mixture, using free and immobilized microbial cells.

## 2. Materials and Methods

### 2.1. Chemicals

Chlorpyrifos (CP) (99% pure) and pentachlorophenol (PCP) (98% pure) were purchased from Sigma-Aldrich Co. (St. Louis, MO, USA). All other chemicals used throughout the study were of analytical grade and were purchased from standard manufacturers.

### 2.2. Microorganisms and Culture Media

Four *Streptomyces* spp. (A2, A5, A11, and M7) isolated from organochlorine pesticides contaminated Argentinian soils and sediments [27, 31], and other two strains (*Streptomyces* spp. AC5 and AC7) isolated from a Chilean soil contaminated with organophosphorus pesticides [6], were used in this study. These actinobacteria were grown as single cultures and combined as different microbial consortia. The first mixed culture was an Argentinian microbial consortium (*Streptomyces* spp. A2-A5-A11-M7), known for its lindane biodegradation potential [32]; the second one was a combination of the two Chilean strains (*Streptomyces* spp. AC5 and AC7), and the third consortium consisted of the six actinobacteria strains together.

Starch-casein medium (SC) was used for antagonism assays, consisting of (g L^−1^): starch, 10.0; casein, 1.0; K_2_HPO_4_, 0.5; agar, 15.0. The pH was adjusted to 7.0 prior to sterilization. 

Minimal medium (MM) was used for growth of the microorganisms and pollutants removal assays. It consisted of (g L^−1^): L-asparagine, 0.5; K_2_HPO_4_, 0.5; MgSO_4_·7H_2_O, 0.2; FeSO_4_·7H_2_O, 0.01 [33]. The same medium was added with agar, 15.0 g L^−1^, for tolerance to toxicity of CP and PCP assay. The pH was adjusted to 7.0 prior to sterilization. 

Tryptic Soy Broth (TSB), containing (g L^−1^): trypticase, 15.0; soy peptone, 3.0; NaCl, 5.0; K_2_HPO_4_, 2.5; glucose, 2.5, was used for the inocula preparation for the immobilization technique. The pH was adjusted to 7.3 ± 0.2 prior to sterilization.

All media were sterilized by autoclaving at 121°C for 15 min.

### 2.3. Antagonism and Tolerance Assays

In order to determine the potential presence of antagonistic effects among the *Streptomyces* spp. strains studied, a modification of Bell et al. [34] technique was used. One *Streptomyces* strain was spread in the center of a Petri dish containing SC medium and faced transversely with the other *Streptomyces* spp. strains. One strain was considered to be antagonistic to the other when a growth inhibition was observed. Thereby, the presence of antagonism among the six studied strains was assessed by considering all possible combinations [32].

To evaluate the tolerance of *Streptomyces* spp. strains to the pesticide mixture (CP and PCP), a qualitative assay was performed using MM agar plates. Rectangular troughs were cut in the centre of the plate and then filled with a filter-sterilized solution of the mixed pesticides (CP and PCP, each at a concentration of 1.66 mg L^−1^). The actinobacteria strains were inoculated by streaking perpendicularly to the troughs. Microbial growth was used as a qualitative parameter of the tolerance to the pesticides mixture. The Petri dishes were incubated at 30°C for seven days. Growth controls were performed using plates without pesticides [35].

### 2.4. Study of the Ability of Pure and Mixed Actinobacteria Cultures to Grow and Remove the Pesticide Mixture

2 g L^−1^ of biomass (wet weight) of the microbial consortia *Streptomyces* spp. A2-A5-A11-M7 and *Streptomyces* spp. AC5 and AC7 and the six pure actinobacteria strains were inoculated in different Erlenmeyer flasks containing 30 mL MM spiked with the pesticides mixture (1.66 mg L^−1^ of each one). Cultures were incubated at 30°C for 72 h on a rotary shaker at 200 rpm and then centrifuged (8,500 ×g, 10 min and 4°C). Ten milliliters of the supernatant were aseptically taken out in each case for residual pesticides determination. Microbial growth was measured as dry weight at 105°C. All experiments were carried out in triplicate, and the results are given as the means.

### 2.5. Removal of Pesticides Mixture by a Free and Immobilized Consortium of Six *Streptomyces* spp. Strains

All *Streptomyces* spp. strains were individually cultured in TSB for 72 h at 30°C and 200 rpm. The cultures were centrifuged at 8,500 ×g for 10 min, and the pellets obtained were then washed with sterile distilled water for the immobilization. For this, the actinobacteria consortium (*Streptomyces* spp. A2-A5-A11-M7-AC5-AC7, each strain at equal proportion), was mixed with a sodium alginate solution, obtaining a final concentration of 7.5% of biomass in the support (w/v, wet weight) of the mixed culture [36]. This mixture was poured into 2% CaCl_2_·2H_2_O solution and incubated at room temperature for 1 h. Then, the beads (3–5 mm diameter) were washed three times with sterile distilled water [37]. All the materials used for the preparation of the entrapped cells were sterilized, and the operations were carried out under sterile conditions.

The immobilized consortium was inoculated into MM containing mixed pesticides (CP and PCP, 1.66 mg L^−1^ of each one) and subsequently incubated for 72 h at 30°C. Samples collected every 24 h were analyzed for residual pesticides concentrations.

For free cell culture assays with all the *Streptomyces* spp. strains together, the methodology used was the same described above (see Section 2.4).

### 2.6. Analysis of Pentachlorophenol and Chlorpyrifos

Supernatant samples of the centrifuged cultures were used to determine residual CP and PCP concentrations. For residual CP concentration determination, 1 mL of each sample was diluted to a volume of 10 mL with distilled water, and then it was extracted twice with 10 mL of hexane. The organic extracts were combined and dehydrated with Na_2_SO_4_. The samples were stored at −20°C before chromatographic analysis. A Shimadzu gas chromatograph GC-2014 equipped with an RTX-5 capillary column (crossbond 5% diphenyl/95% dimethyl polysiloxane, 30 m, 0.32 mm i.d., film thickness 0.25 *μ*m), and NPD detector was used. The injection and detector temperatures were set at 280°C and 300°C, respectively. The oven temperature program began at 90°C for 1 min, increased to 180°C at 15°C/min, then increased to 240°C at 5°C/min, and finally increased to 280°C at 15°C/min. The obtained data were analyzed with the program GC Solution Version 2.30.00 (GC Solution Analysis Copyright 2000–2004 Shimadzu) [6]. The retention time for CP was 12.5 min. The recovery of CP in the liquid medium was 90%.

Residual PCP concentration was determined with an HPLC equipped with a Merck-Hitachi L-7100 pump, a Rheodyne 7725 injector with a 20-*μ*L loop, a Merck-Hitachi L-7455 diode array detector operating at 215 nm and a Hitachi D-7000 data processor. A LiCHrospher 60 RP select B 250 × 4 mm column of 5 *μ*m particle size with a LichroCART 4-4 guard column (Merck) was used. The mobile phase consisted of acetonitrile and phosphoric acid (1% aqueous solution) 1 : 1 (v/v) with a flow rate of 1 mL min^−1^. In these operative conditions, PCP retention time was 12 min [38]. Method calibration and quantification was performed by the pure reference standard (0.05–5 mg L^−1^). The recovery of PCP ranged from 97% to 100%.

### 2.7. Statistical Analysis

All the results are the average of three replicates per sample. One-way analysis of variance (ANOVA) and Tukey test were performed to test the significant differences among treatments. When significant differences were found, Tukey post-test was used to separate the effects among treatments. Tests were considered significantly different at *P* < 0.05. Professional versions of Infostat and Statistic 6.0 software were used.

## 3. Results and Discussion

### 3.1. Antagonism and Tolerance Assays

**Figure 1:**
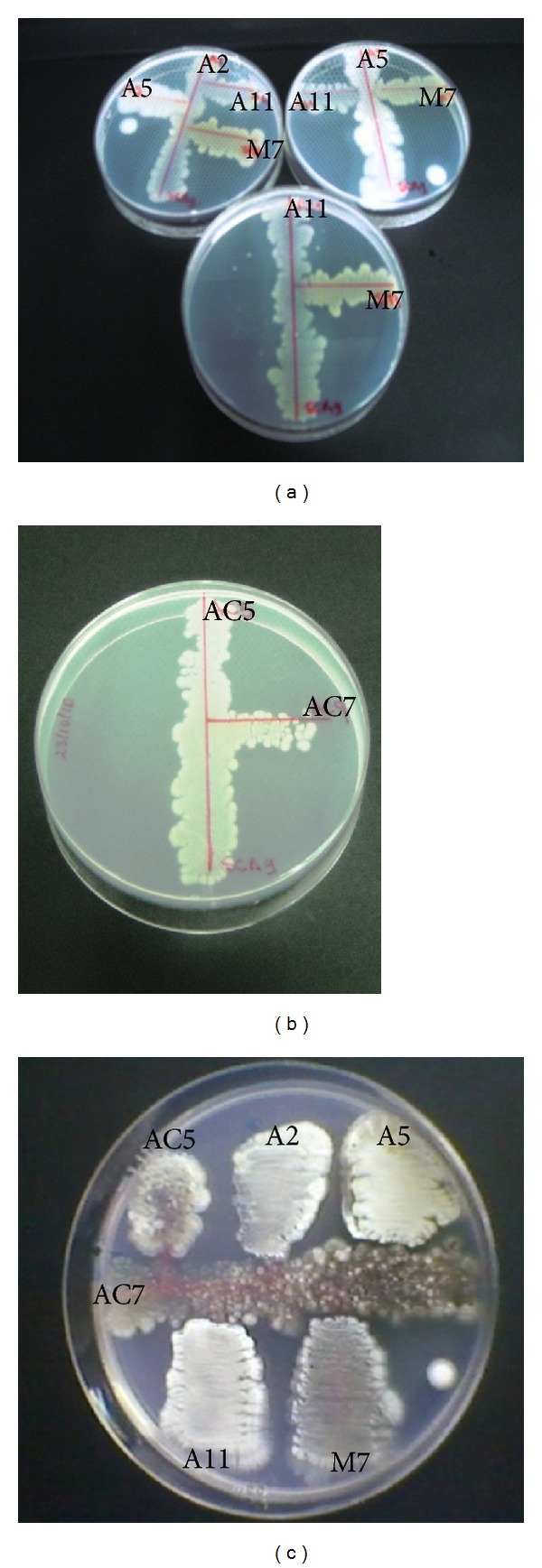
Antagonism assays among: (a) *Streptomyces* spp. A2, A5, A11, and M7; (b) *Streptomyces* spp. AC5 and AC7; and (c) the six *Streptomyces* spp. strains.

In order to formulate mixed cultures, all the studied actinobacteria were assayed to determine the presence of antagonistic effects among them. The antagonistic phenomenon is a common event showed in a mixed microbial population [39]. In case of *Streptomyces* spp. strains isolated from Argentinean and Chilean environments, no antagonistic effect on their individual growth was observed (Figure 1), which suggests that all the strains could be cultured together as a consortium. In contrast, Thouand et al. [40] observed the presence of antagonistic relations in a mixed microbial population, which exerted a negative impact on the ability of oil degradation in liquid systems. In fact, van Hamme et al. [41] showed the presence of antagonistic relations in bacterial populations within a mixed community, due to the metabolites production capable of killing or inhibiting the growth of other populations.

**Figure 2:**
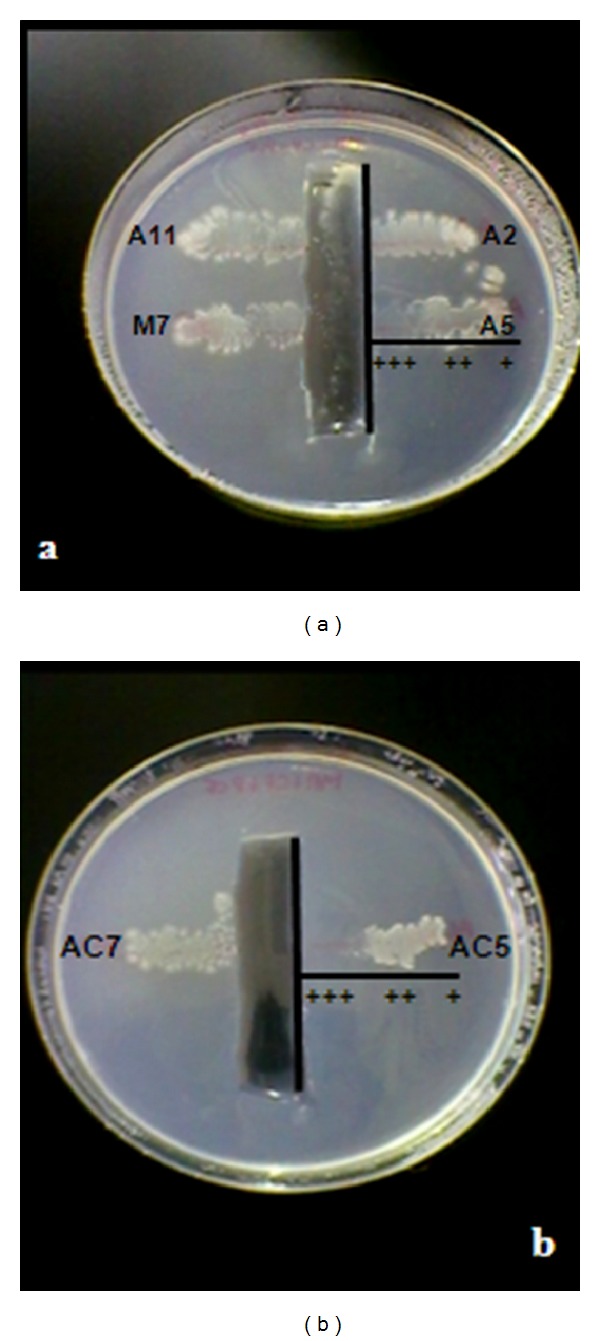
Tolerance assays of the studied actinobacteria to the mixture of CP and PCP (1.66 mg L^−1^ of each one). (a) *Streptomyces* spp. A2, A5, A11, and M7. (b) *Streptomyces* spp. AC5 and AC7. (+++) Abundant growth: highly tolerant, (++) moderate growth: tolerant, (+) scarce growth: lowly tolerant, and (−) no growth: not tolerant.

Although it have been described actinobacteria capable of tolerating and/or degrading CP or PCP [6, 28, 42, 43], it was necessary to evaluate the ability of the actinobacteria to tolerate the pesticides mixture. Thereby, when the actinobacteria tolerance to the mixed pesticides was tested, it was observed that four of them (*Streptomyces* spp. A2, A11, M7 and AC7) showed a high degree of tolerance to the toxic mixture, while the other two strains (*Streptomyces* spp. A5 and AC5) presented moderate tolerance, based on qualitative analyses taking into account the degree of growth of each strain in the surroundings of the mixture of pesticides (Figure 2). This experimental technique was used previously for systematic screening on heavy metals resistance and organochlorine pesticides tolerance by actinobacteria [31, 44]. The results presented here would indicate that the pesticides mixture concentration used were not toxic for these actinobacteria strains under the evaluated experimental conditions.

### 3.2. Microbial Growth and Removal of Pesticides Mixture by Pure and Mixed Actinobacteria Cultures

**Figure 3:**
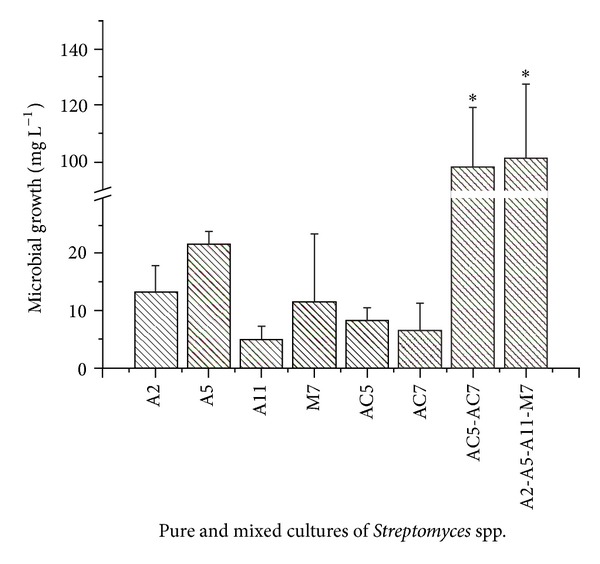
Microbial growth of pure and mixed cultures of *Streptomyces* spp. in MM contaminated with a mixture of chlorpyrifos (CP) and pentachlorophenol (PCP). Bars showing asterisk indicate they were significantly different to all others (*P* < 0.05, Tukey post-test).

Based on obtained results (Section 3.1) and previous studies, which describe the actinobacteria abilities to use compounds with chlorine atoms in their molecules as carbon source [6, 27, 28, 43, 45], the growth of the strains as pure and mixed cultures in liquid MM supplemented with the pesticides mix (CP + PCP) was evaluated. Microbial biomass (dry weight) of the pure cultures ranged between 5 and 21.7 mg L^−1^, whereas the growth of the mixed cultures was significantly higher (*P* < 0.05), reaching a biomass of 98.3 mg L^−1^ for the mixed culture of *Streptomyces* spp. AC5-AC7 and 101.67 mg L^−1^ for *Streptomyces* spp. A2-A5-A11-M7 (Figure 3). In absence of the pesticides mixture in the culture medium, no growth was detected. The highest biomass production of mixed cultures could be explained due to a metabolic action complementary among actinobacteria in the consortia, which make them capable of allowing the most efficient use of these pesticides as carbon source. In previous studies, Yang et al. [46] observed a high atrazine mineralizing efficiency when a mixed culture* of Klebsiella* sp. A1 and *Comamonas* sp. A2 was used. However, when these authors used pure cultures, they obtained no or poor growth and no or less atrazine degrading ability. In the present study, the results showed an increase of the biomass when increasing the number of strains in the culture medium, demonstrating that there is no inhibition of growth by the presence of pesticides or antagonism among strains.

**Figure 4:**
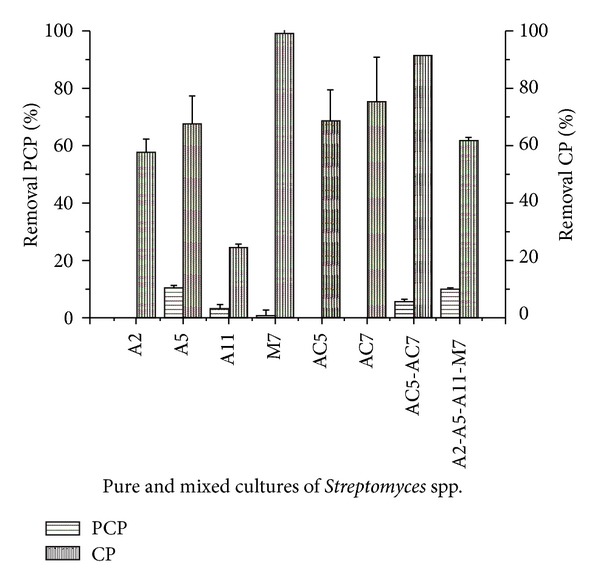
Removal percentages of pentachlorophenol (PCP) and chlorpyrifos (CP) in pure and mixed cultures of *Streptomyces* spp. in minimal medium contaminated with the pesticides mixture.

The pesticide removal abilities of the pure and mixed cultures were determined by analyzing CP and PCP residual concentrations. It was observed that *Streptomyces* sp. A5 and the mixed culture *Streptomyces* spp. A2-A5-A11-M7 presented similar PCP removal percentages (10.6 and 10.1%, resp.); the mixed culture *Streptomyces* spp. AC5-AC7 only removed 6% of PCP; instead *Streptomyces* spp. A2, AC5, and AC7 in pure cultures did not show the ability to remove PCP (Figure 4). Compared to most of the pure cultures, removal of PCP was significantly enhanced (*P* < 0.05) when these strains were grown in a coculture. Similar enhanced degradation has been observed in many studies of different pesticide-degrading consortia. For instance, Sørensen et al. [47] reported an enhancement of 59% in the mineralization of isoproturon when *Sphingomonas* sp. SRS2 was grown in coculture with SRS1 strain, rather than pure. Other coculture was able to mineralize a 62% of the added diuron (10 mg L^−1^) in a mineral medium due to the cooperative degradative capacities of *Arthrobacter globiformis* strain D47 and *Variovorax* sp. strain SRS16, since neither strain D47 nor strain SRS16 was capable of performing extensive mineralization of the herbicide in pure culture [48]. In natural environments, microorganisms are heterogeneously distributed and possibly occur in multispecies rather than single-species communities [47]. Close proximity within the community may synergistically improve the metabolism of organic pollutants introduced into the environment [47]. In these studies, neither strain *Streptomyces* sp. AC5 nor strain *Streptomyces* sp AC7 conclusively removed PCP in MM but combined the constructed two-member consortium removed approximately 6% of PCP, demonstrating that synergistic interactions between both strains may be involved in the degradation of PCP.

All the pure and mixed actinobacteria cultures were able to remove CP, reaching removal percentages greater than PCP removal. The strain *Streptomyces* sp. M7 in pure culture showed the best CP removal capability (99.2%) but the mixed culture *Streptomyces* spp. AC5-AC7 also showed high ability for CP removal (91.52%) (Figure 4). In previous studies, Krishna and Philip [3] observed great differences in the removal efficiency of three toxic compounds in a submerged soil system contaminated with a mixture of pesticides (carbofuran, lindane, and methyl parathion at a final concentration of 2 mg g^−1^ of soil), where carbofuran degradation was maximum whereas minimum degradation was observed for lindane, both in the soil phase and in the liquid phase. They also found that in the mixture of these pesticides, the degradation efficiency was minor than the efficiency detected in systems contaminated with one pesticide at a time. This phenomenon was attributed to the lower number of microorganisms available to degrade specific individual pesticides. In the present work, this phenomenon could also explain the low PCP removal obtained in comparison to CP removal, although further studies are required to prove it. On the other hand, Buono et al. [49], who studied the toxic effects of pesticides of current use (PCP, azinphos-methyl (AZM) and CP) on the development of *Paracentrotus lividus* embryos, demonstrated that the most toxic pesticides were PCP and AZM at EC50 (median toxic effect concentration 50%) level. They also observed that PCP toxic effects were not significant at concentrations below 0.03 mg L^−1^, but at higher concentrations, such as 0.3 mg L^−1^; the effects were significant. In this work, the PCP concentration was approximately 5.5 times higher than 0.3 mg L^−1^, which could be another reason for the minor removal of this compound. On the contrary, Matamoros et al. [50], in a study pertaining to behavior of organic pollutants in constructed wetlands, found that PCP removal efficiency was higher (>90%) than CP removal (80%–90%), starting with an initial concentration of 2.5 mg L^−1^ of each pollutant. Although in the present study, the CP was the pesticide with the highest removal percentages from the mixture.

All *Streptomyces *strains studied at the present work have been previously exposed to different chlorinated pesticides. Thus, these microorganisms could have the enzymatic ability to release chloride ions, favoring its degradation. The cross-adaptation phenomenon suggests that one pesticide may be rapidly degraded in soil in which it has never been applied; provided that the same soil had been previously exposed to a pesticide belonging to the same chemical group [51]. This could explain removal percentages obtained for both pesticides, PCP and CP, for strains as *Streptomyces* sp. A5 or *Streptomyces* sp. M7.

Another actinobacteria strain, *Kocuria* sp. CL2 isolated from secondary sludge of pulp and paper mill, able to use PCP as the sole source of carbon and degrade this pesticide, had been reported [28]. 

### 3.3. Removal of Pesticides Mixture by a Consortium of Six *Streptomyces* spp. Strains Free and Immobilized

Different researchers have demonstrated that cell immobilization techniques can significantly increase the removal efficiency of different pesticides compared with free cells [36, 52]. Thus, removal of a pesticides mixture (CP and PCP) by a six actinobacteria consortium, either free or immobilized in alginate beads, was compared. 

The results revealed that CP removal was higher than PCP removal, following the same trend observed in previous assays (see Section 3.2), both in free and immobilized mixed cultures. CP removal percentages were 40.17 and 71.05 for free and immobilized cells, respectively. For PCP removal, the obtained values were 5.24% and 14.74% for the free and immobilized systems, respectively (Table 1). Thus, it is evident the increase of the removal of both pesticides by using the microbial immobilization technique. A possible reason for this could be that the alginate beads allow the optimal diffusion of contaminants [53–55] and besides; the support could be also acting as a protection for the cells against the detrimental effects of the surrounding medium such as pH and toxic substances, also enhancing the degrading ability of the cells [56, 57]. In fact, several researchers have demonstrated that calcium alginate immobilization improved the removal of toxic compounds. For instance, a *Pseudomonas* strain immobilized in calcium alginate mineralized a 50% more of phenol than free cells under the same conditions [58]. Also, Yañez-Ocampo et al. [52] studied the removal of two organophosphate pesticides by a bacterial consortium, and they obtained a percentage of methyl parathion removed 31% higher when the consortium was immobilized in alginate beads, compared with a suspension culture. 

On the other hand, the pesticide sorption phenomenon to the alginate support was registered for both compounds. However, analysis of CP removal showed that the phenomenon of sorption was gradual, increasing up to 72 h, whereas for PCP it remained almost stable from 24 h until the end of the assay (data not shown). The sorption percentage was higher for CP (60.34% ± 0.42%) than for PCP (5.97% ± 2.41%) (data not shown), although the pesticides removal percentages observed by using alginate beads with or without microorganisms were significantly different (*P* < 0.05), evidencing the microbial activity. The sorption of different compounds, such as dyes and pesticides, on different immobilization supports was also reported by other researchers [36, 59].

Furthermore, when comparing the ability of removing the pesticides among all the free cell cultures, it might be concluded that the use of the six *Streptomyces* strains together did not present the best percentages of removal of the pesticides. These results are similar to those obtained by Fuentes et al. [32] in which mixed cultures consisting in two, three, and four strains improved the lindane removal compared with pure cultures, whereas combinations of five and six strains were not efficient for the removal of the pesticide from the culture medium.

**Figure 5:**
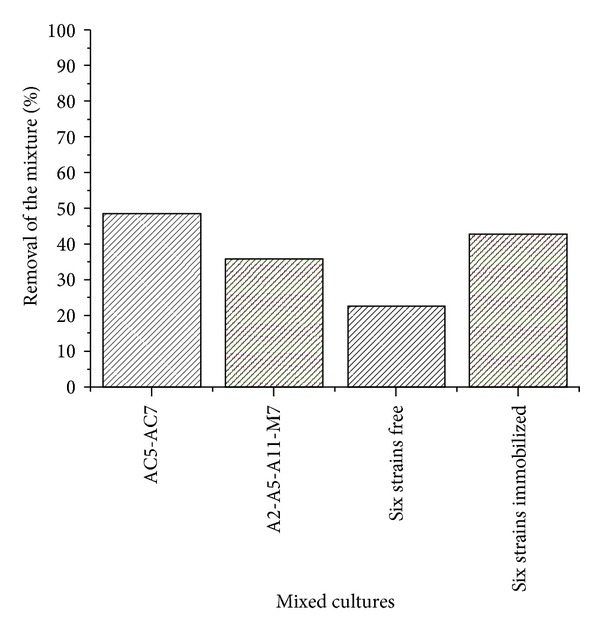
Removal percentages of the mixture (CP + PCP) for mixed cultures of *Streptomyces* spp. strains (A2, A5, A11, M7, AC5, AC7) as free and immobilized cells.

Moreover, the analysis of the removal percentage of the pesticides mixture, calculated as the average between the removal percentages of CP and PCP for the mixed cultures, showed that the double consortium (*Streptomyces* spp. AC5-AC7) and the mixed culture of the six strains immobilized (*Streptomyces* spp. A2-A5-A11-M7-AC5-AC7) were the consortia with higher ability to removal the toxic mixture (48.64% and 42.90%, resp.) (Figure 5).

## 4. Conclusions

Six *Streptomyces* spp. strains were able to tolerate a mixture of PCP and CP and did not show antagonistic effects among them. These strains were also able to grow and remove mixed pesticides, in pure as well as in mixed cultures. The immobilization of the cells allowed an increase of the removal of both pesticides. Our results reveal that *Streptomyces* strains could be used in mixed cultures and in immobilized systems as a potential tool for remediation of environments contaminated with multiple xenobiotics.

## Figures and Tables

**Table 1:** Pesticides removal percentages of the six actinobacteria strains culture in free and immobilized cells systems.

Sixfold culture	PCP removal (%)	CP removal (%)
Free cells	5.24 ± 0.56	40.17 ± 1.79
Immobilized cells	14.74 ± 4.73	71.05 ± 0.88
